# Evaluation of the FPMC respiratory panel for detection of respiratory tract pathogens in nasopharyngeal swab and sputum specimens

**DOI:** 10.1186/s12985-024-02430-x

**Published:** 2024-07-11

**Authors:** Li Xue, Jianhong Zhu, Ke Lei, Zeshi Liu, Yiwei Tang, Bo Zhong, Ning Gao, Chaoliang Xiong, Jing Lei, Ying Tian, Weixiao Zhou, Nan Feng, Xue Zhang, Dong Chen, Jing Li, Yan Geng

**Affiliations:** 1 Department of Clinical Laboratory, The Second Affiliated Hospital of Xi’an Jiaotong University, Xi’an, Shaanxi China; 2 Cepheid, Danaher Diagnostic Platform, Shanghai, China; 3 Department of Pediatrics, The Second Affiliated Hospital of Xi’an Jiaotong University, Xi’an, Shaanxi China

**Keywords:** Fluorescent probe melting curve PCR, Respiratory pathogen, Molecular detection

## Abstract

**Objectives:**

The performance of the new Respiratory Pathogen panel (fluorescent probe melting curve, FPMC) for the qualitative detection of 12 organisms (chlamydia pneumoniae, mycoplasma pneumoniae, adenovirus, influenza A virus, influenza B virus, parainfluenza virus, rhinovirus, etc.) was assessed.

**Methods:**

Prospectively collected nasopharyngeal swab (NPS) and sputum specimens (*n* = 635) were detected by using the FPMC panel, with the Sanger sequencing method as the comparative method.

**Results:**

The overall percent concordance between the FPMC analysis method and the Sanger sequencing method was 100% and 99.66% for NPS and sputum specimens, respectively. The FPMC testified an overall positive percent concordance of 100% for both NPS and sputum specimens. The FPMC analysis method also testified an overall negative percent concordance of 100% and 99.38% for NPS and sputum specimens, respectively.

**Conclusions:**

The FPMC analysis method is a stable and accurate assay for rapid, comprehensive detecting for respiratory pathogens.

**Supplementary Information:**

The online version contains supplementary material available at 10.1186/s12985-024-02430-x.

## Introduction

Respiratory tract infection (RTI) is one of the most important causes for extensive morbidity and mortality among patients worldwide [1]. Different pathogens could induce similar symptoms and signs of RTI, which is mainly characterized by upper respiratory infections such as rhinitis, pharyngitis, laryngitis, tonsillitis, etc. [2]. And some patients with RTI show severe symptoms of lower respiratory infections including tracheitis, bronchitis and pneumonia [3]. It has been demonstrated that most acute respiratory tract infections are induced by viruses including respiratory syncytial virus, adenovirus, influenza A and B viruses, parainfluenza virus, and so on [4]. Respiratory virus infection is one of the most common diseases for people in all age groups [5]. Notably, treatment method, curative effect and disease course vary between patients with RTI induced by different pathogens [6]. Therefore, accurate and timely etiological analysis is not only essential for diagnosis of RTI, but also the basis for reasonable selection of appropriate therapeutic regimen [7, 8]. And it is also urgent to develop new methods of rapid detection of respiratory viruses.

Single-tube multiplex fluorescent probe melting curve (FPMC) technology (four channel detection: Fam, Vic, Rox, Cy5) and fusion curve analysis employed in the new detection method is evaluated in this study. Detection using this new method covers a wide range of 12 kinds of pathogen nucleic acids, including *chlamydia pneumoniae*, *mycoplasma pneumoniae*, adenovirus, influenza A virus, influenza B virus, parainfluenza virus (types 1, 2, 3 and 4), rhinovirus, respiratory syncytial virus, Boca virus, metapneumovirus, coronavirus (229E, hku1, nl63 and OC43), or novel coronavirus. Specifically, three channels (Fam, Vic and Rox) were employed to detect the target pathogen, and pathogens in a sample are identified according to the cycle threshold (CT) values in each channel during the process of PCR amplification and the corresponding change rate of peak height within a range of the melting temperatures of the targeted pathogens. Additionally, Cy5 channel was used for detecting the endogenous internal standard in order to monitor the quality of samples and the accuracy of experimental processes.

To have insight into the novel diagnostic assay for clinical application, we here provide important evidence comparing the diagnostic accuracy of the FPMC analysis method and Sanger sequencing method for the detection of RTI.

## Methods

### Study population

This study included 635 patients of all ages and both genders showing with signs and/or symptoms of respiratory tract infection such as cough, nasal congestion, runny nose, sore throat, loss of smell or taste, dyspnea, lung related diseases, etc. These patients were all from the second affiliated hospital of Xi’an Jiaotong University from March 2021 through August 2021. Respiratory specimens including nasopharyngeal swab (NPS) and sputum specimens were collected. This study involves human participants and was approved by the Research Committee of Human Investigation of the Second Affiliated Hospital, Xi’an Jiaotong University.

### FPMC analysis method

The new test was conducted by Fluorescent PCR melting curve method in accordance with instructions of the manufacturer. The assay consists of several steps including nucleic acid extraction, reverse transcription, DNA amplification, and results analyses. For sputum samples with high viscosity, it was required to perform preprocessing method by adding the reagents to liquefy these sputum samples. Then approximately 200 ul of specimen was processed using the BaoChuang Biotech total nucleic acid extraction kit. PCR testing was conducted according to specifications of the manufacturer. The analysis software could provide functions of results interpretation. Generally, each target gene of a sample in a valid test is reported as “detected” or “not detected.” If the internal control gene of a sample is not detected, the software automatically shows an invalid result for all the panel genes of the corresponding sample. The new FPMC analysis method has corresponding positive controls for all the tested viruses in the assay.

### Sanger sequencing method

PCR products were purified and then performed Sanger sequencing using ABI-3730xl DNA Sequencer. Sanger sequencing was conducted by Liuhe Huada Gene Technology Co., Ltd. Guangzhou Branch. Sanger sequencing reaction system consists of DNA templates, DNA polymerase enzymes, primers, a mixture of the 4 deoxynucleotide triphosphates (dNTPs: dATP, dGTP, dCTP, dTTP) and chain-terminating dideoxynucleotide triphosphates (ddNTPs: ddATP, ddGTP, ddCTP, ddTTP). Due to the random incorporation of ddNTPs at each position of the PCR product, and the lack of a 3 ‘- terminal hydroxyl group on the deoxyribose, the elongation reaction of DNA strands terminated at that position where the ddNTPs are incorporated. As a result, a series of DNA fragments of varying lengths can be synthesized with a common primer at the 5 ‘end and a dideoxy base at the 3’ end in this reaction system. Then capillary electrophoresis can be applied for separating DNA fragments by the length and the sequence of base arrangement in target fragments can be read sequentially based on the fluorescence signal of the terminating base. Data was analyzed by using Sequencing Analysis 5.2 software. The primers used to amplify the segment for sequencing were shown as Table S1.

### Statistical methods

Positive percent agreement (PPA), negative percent agreement (NPA), and overall percent agreement (OPA) with results of FPMC analysis assay were determined by comparing those of the Sanger sequencing method. The PPA was shown as 100 × number of true positive (TP) / (number of TP + number of false negative (FN)), the NPA was shown as 100 × number of true negative (TN)/ (number of TN + number of false positive (FP)), and OPA was shown as 100 × (number of TP + number of TN)/(number of TP + number of TN + number of FP + number of FN). The two-sided 95% score confidence interval (CI) was calculated for PPA, NPA, and OPA. Statistical analyses were conducted by using SPSS 19.0.

## Results

### Patient demographics

A total of 635 prospectively gathered samples were qualified to test using the FPMC analysis method. Around 67% of specimens were NPS, and 33% were sputum. Generally, the study included samples from more male subjects than the females (66% [422/635] and 34% [213/635], respectively). A total of 18% of the samples were from children aged 5 years and under, 13% were from those aged 6 to 21 years, 34% were from adults aged 22 to 59 years, and 35% were from adults over the age of 60 years. Demographic information for the 635 clinical specimens is provided in Table 1.

**Table 1:** Subject demographics

Parameter	Sample type	
	NPS (*n* = 344)	Sputum (*n* = 291)
No. (%) of subjects of gender		
Male	223 (64.8)	199 (68.4)
Female	121 (35.2)	92 (31.6)
No. (%) of subjects in age group		
≤ 5 years	89 (25.9)	25 (8.59)
6–21 years	74 (21.5)	7 (2.41)
22–59 years	116 (33.7)	100 (34.4)
≥ 60 years	65 (18.9)	159 (54.6)

NPS, nasopharyngeal swab

### Distribution of pathogens by FPMC assay

The overall rate of pathogens infection was 56.5% in the study subjects. Among 359 positive patients, 319 (88.9%, 319/359) patients had a single pathogen, 37 (10.3%, 37/359) patients had two pathogens, and 3 (0.8%, 3/359) patients had three pathogens. Human rhinovirus was the most prevalent pathogen (35.9%, 129/359), followed by coronavirus (20.3, 73/359), parainfluenza virus (18.1%, 65/359), adenovirus (10.3%, 37/359). Others were as below: respiratory syncytial virus (9.5%, 34/359), influenza B virus (8.9%, 32/359), human bocavirus (5.6%, 20/359), *mycoplasma pneumoniae* (2.8%, 10/359), *chlamydia pneumoniae* (0.3%, 1/359), human metapneumovirus (0.3%, 1/359) (Table 2). As shown in Table 2, the difference was detected among groups with different ages. Higher proportions of human rhinovirus were identified in the group aged 22 to 59 years. Coronavirus were also shown higher proportions in the group aged 22 to 59 years. Parainfluenza virus was found to be higher proportions in the group aged 5 years and under compared with other groups. Only two pathogens including human rhinovirus and influenza B virus were found in both NPS and sputum species of group aged 6 to 21 years.

**Table 2:** Prevalence of FPMC assay-detected analytes stratified by age group

	Prevalence of analytes in species of indicated subject group									
	Overall		≤ 5 years		6–21 years		22–59 years		≥ 60 years	
	(*n* = 359)		(*n* = 80)		(*n* = 54)		(*n* = 111)		(*n* = 114)	
**Target**	**NPS** **No. (%)**	**Sputum** **No. (%)**	**NPS** **No. (%)**	**sputum** **No. (%)**	**NPS** **No. (%)**	**Sputum** **No. (%)**	**NPS** **No. (%)**	**Sputum** **No. (%)**	**NPS** **No. (%)**	**Sputum** **No. (%)**
Influenza A virus	0 (0.00)	0 (0.00)	0 (0.00)	0 (0.00)	0 (0.00)	0 (0.00)	0 (0.00)	0 (0.00)	0 (0.00)	0 (0.00)
Influenza B virus	17 (4.94)	15 (5.15)	3 (3.37)	0 (0.00)	8 (10.81)	1 (14.29)	5 (4.31)	1 (1.00)	1 (1.54)	13 (8.18)
Respiratory syncytial virus	14 (4.07)	20 (6.87)	8 (8.99)	0 (0.00)	0 (0.00)	0 (0.00)	2 (1.72)	10 (10.00)	4 (6.15)	10 (6.29)
Adenovirus	19 (5.52)	18 (6.19)	5 (5.62)	2 (8.00)	5 (6.76)	0 (0.00)	6 (5.17)	5 (5.00)	3 (4.62)	11 (6.92)
metapneumovirus	0 (0.00)	1 (0.34)	0 (0.00)	0 (0.00)	0 (0.00)	0 (0.00)	0 (0.00)	0 (0.00)	0 (0.00)	1 (0.63)
Human bocavirus	7 (2.03)	13 (4.47)	4 (4.49)	1 (4.00)	0 (0.00)	0 (0.00)	2 (1.72)	5 (5.00)	1 (1.54)	7 (4.40)
Mycoplasma pneumoniae	1 (0.29)	9 (3.09)	0 (0.00)	1 (4.00)	1 (1.35)	0 (0.00)	0 (0.00)	2 (2.00)	0 (0.00)	6 (3.77)
Chlamydia pneumoniae	0 (0.00)	1 (0.34)	0 (0.00)	0 (0.00)	0 (000)	0 (0.00)	0 (0.00)	1 (1.00)	0 (0.00)	0 (0.00)
Human rhinovirus	93 (27.03)	36 (12.37)	30 (33.71)	2 (8.00)	24 (32.43)	2 (28.57)	27 (23.28)	13 (13.00)	12 (18.46)	19 (11.95)
Coronavirus	64 (18.60)	9 (3.09)	13 (14.61)	0 (0.00)	14 (18.92)	0 (0.00)	25 (21.55)	3 (3.00)	12 (18.46)	6 (3.77)
Parainfluenza virus	42 (12.21)	23 (7.90)	28 (31.46)	2 (8.00)	4 (5.41)	0 (0.00)	6 (5.17)	7 (7.00)	4 (6.15)	14 (8.81)
novel coronavirus	0 (0.00)	0 (0.00)	0 (0.00)	0 (0.00)	0 (0.00)	0 (0.00)	0 (0.00)	0 (0.00)	0 (0.00)	0 (0.00)

FPMC, fluorescent probe melting curve; NPS, nasopharyngeal swab

The distribution of multi-pathogens combination was described in Table S2. A total of 40 multi-pathogens subjects were detected with 17 different combination types. The combination of human rhinovirus plus coronavirus or parainfluenza virus was the most common form. Moreover, human rhinovirus was observed in the majority of multi-organism-positive patients.

### Comparative analysis and discrepancy investigation

A summary of comparative performances of the FPMC analysis method and the sequencing method is provided in Tables 3 and 4. PPA and NPA were calculated with regard to the sequencing method along with 95% CI. The two assays had an overall concordance of 100% (κ = 1.0000) and 99.66% (κ = 0.9930) for NPS and sputum, respectively. The total PPA with respect to the sequencing method was 100% for both NPS and sputum, and the total NPA with regard to the sequencing method was 100% and 99.38% for NPS and sputum, respectively.

**Table 3:** Performance summary of the FPMC Respiratory Panel for nasopharyngeal swab specimens

	PPA			NPA		
Target	TP/TP + FN	%	95% CI	TN/TN + FP	%	95% CI
Influenza B virus	17/17	100.00	81.57–100.00	327/327	100.00	98.84–100.00
Respiratory syncytial virus	12/12	100.00	75.75–100.00	330/332	99.40	97.83–99.83
Adenovirus	19/19	100.00	83.18–100.00	325/325	100.00	98.83–100.00
Metapneumovirus	/	/	/	344/344	100.00	98.90–100.00
Human bocavirus	6/6	100.00	60.97–100.00	337/338	99.70	98.34–99.95
Mycoplasma pneumoniae	1/1	100.00	20.65–100.00	343/343	100.00	98.89–100.00
Chlamydia pneumoniae	/	/	/	344/344	100.00	98.89–100.00
Human rhinovirus	92/92	100.00	95.99–100.00	251/252	99.60	97.79–99.93
Coronavirus	64/64	100.00	94.34–100.00	280/280	100.00	98.65–100.00
Parainfluenza virus	42/42	100.00	91.62–100.00	302/302	100.00	98.74–100.00

FPMC, fluorescent probe melting curve; PPA, positive percent agreement; NPA, negative percent agreement; TP, true-positive result; FN, false-negative result; TN, true-negative result; FP, false-positive result

**Table 4:** Performance summary of the FPMC Respiratory Panel for sputum specimens

	PPA			NPA		
Target	TP/TP + FN	%	95% CI	TN/TN + FP	%	95% CI
Influenza B virus	15/15	100.00	81.57–100.00	276/276	100.00	98.84–100.00
Respiratory syncytial virus	20/20	100.00	83.89–100.00	271/271	100.00	98.60–100.00
Adenovirus	18/18	100.00	82.41–100.00	273/273	100.00	98.61–100.00
Metapneumovirus	1/1	100.00	20.65–100.00	290/290	100.00	98.69–100.00
Human bocavirus	11/11	100.00	74.12–100.00	278/280	99.29	97.43–99.80
Mycoplasma pneumoniae	9/9	100.00	70.08–100.00	282/282	100.00	98.66–100.00
Chlamydia pneumoniae	1/1	100.00	20.65–100.00	290/290	100.00	98.69–100.00
Human rhinovirus	35/35	100.00	90.11–100.00	255/256	99.61	97.82–99.93
Coronavirus	9/9	100.00	70.08–100.00	282/282	100.00	98.66–100.00
Parainfluenza virus	22/22	100.00	85.13–100.00	268/269	99.63	97.92–99.93

FPMC, fluorescent probe melting curve; PPA, positive percent agreement; NPA, negative percent agreement; TP, true-positive result; FN, false-negative result; TN, true-negative result; FP, false-positive result

The total percent agreement between the method and the sequencing method was 100% for influenza B virus, adenovirus, coronavirus, *mycoplasma pneumoniae*, *chlamydia pneumoniae* and metapneumovirus. The method showed an NPA of ≥ 99% for other pathogens, with 95% CI of ≥ 97%. For detecting respiratory syncytial virus in NPS and parainfluenza virus in sputum, the NPA was 99.40% (95% CI, 997.83–99.83%) and 99.63% (95% CI, 97.92–99.93%), respectively. The method demonstrated a NPA of 99.70% (95% CI, 98.34–99.95%) in NPS and 99.29% (95% CI, 97.43–99.80%) in sputum for human bocavirus. For human rhinovirus, the NPA was 99.60 (95% CI, 97.79–99.93%) in NPS and 99.61% (95% CI, 97.82–99.93%) in sputum.

Discordance results show that the FPMC analysis method had a high consistency with the sequencing method (Table 5). In Table 5, samples with targets that were detected by the FPMC method but not by the sequencing method are regarded as false-positive (FP). Samples with targets not detected by the FPMC method but by the sequencing method are regarded as false-negative (FN). The specific pathogens in a total of 8 samples were not detected by the sequencing method. The FP results of 8 samples include 2 for respiratory syncytial virus, 3 for human bocavirus, 2 for human rhinovirus, and 1 for parainfluenza virus.

**Table 5:** Discordance results from comparison between the FPMC assay and the sequencing method

Target	No. of FPMC ^+^ / Sequencing ^+^ results (TP)	No. of FPMC ^+^ / Sequencing ^−^ results (FP)	No. of FPMC ^−^ / Sequencing ^+^ results (FN)	No. of FPMC ^−^ / Sequencing ^−^ results (TN)
Influenza B virus	32	0	0	603
Respiratory syncytial virus	32	2	0	601
Adenovirus	37	0	0	598
Metapneumovirus	1	0	0	634
Human bocavirus	17	3	0	615
M.pneumoniae	10	0	0	625
Chlamydia pneumoniae	1	0	0	634
Human rhinovirus	127	2	0	506
Coronavirus	73	0	0	562
Parainfluenza virus	64	1	0	570

FPMC, fluorescent probe melting curve; TP, true-positive result; FP, false-positive result; FN, false-negative result; TN, true-negative result

## Discussion

In this study, the performance characteristics of the new FPMC analysis method were evaluated by assessing agreement with results of the sequencing method, a generally accepted standard method. The FPMC analysis method could provide the accessible options for clinical laboratories focusing on the rapid diagnosis of respiratory tract infections using multiplexed molecular method on NPS and sputum samples. Our results demonstrate that the FPMC analysis method provides a large panel of both viral and other respiratory pathogens in a simple design pattern with a relatively less time to obtain results.

Viruses always contribute to the etiology of RTIs and are regarded as the leading cause of several severe RTIs [9]. Our study found that human rhinovirus was the most common in RTI, and that human rhinovirus was the only identified virus in a substantial part of patients. Human rhinovirus are traditionally regarded as be linked to upper respiratory tract infection [10], otitis media [11], and sinusitis [12]. Recently human rhinovirus has been increasingly recognized as one of the lower respiratory tract pathogens, especially in patients with asthma [13], infants [14], elderly patients [15], and immunocompromised adults [16] with the emerging application of PCR assays for detection of respiratory viruses in clinical laboratories. It was reported that human rhinovirus is the etiology of one-half to two-thirds of common colds, which can also bring about considerable economic burden in respect of medical visit [17, 18]. Given the frequency and consequence of human rhinovirus infections, effective control of the virus by prevention and treatment would make significant impacts on public health. Moreover, atypical respiratory pathogens such as *mycoplasma pneumoniae* and *chlamydia pneumoniae* have become a public health problem in many countries of the world [19]. There are some reports depicting that symptoms of atypical respiratory infections is identical to viral respiratory infections and that co-infection of atypical respiratory pathogens with other viruses could be also detected [20].

Recently, co-infection with multiple pathogens is growingly acknowledged as be both common and important for disease manifestation. Our study showed that 37 patients had two pathogens, with human rhinovirus plus parainfluenza virus or coronavirus being the most types. Additionally, three patients were found to have three organisms including human rhinovirus, coronavirus and parainfluenza virus/adenovirus. The treatment may be more difficult for patients with co-infection of several organisms relative to those with infection of only one organism. Previous studies have shown that co-infections of viruses with other pathogens were detected in patients with RTIs [21] and this phenomenon was also found in 3 specimens with *mycoplasma pneumoniae* plus viruses like adenovirus, parainfluenza virus or influenza B virus.

At present, the main methods for detecting viruses include virus isolation and culture, electron microscopy, direct immunofluorescence (IF), indirect IF, alkaline phosphatase and anti-alkaline phosphatase bridge linked enzyme labeling (AP-AAP), biotin streptavidin peroxidase, enzyme-linked immunosorbent assay, and molecular biology methods like multiple reverse transcription polymerase chain reaction (mRT-PCR), nested PCR and nucleic acid hybridization, multiple real-time PCR, gene chip technology, suspension array technology etc. Virus isolation and culture is a most classic method, by which the existence and type of viruses can be objectively exhibited. However, the long culture cycle and low sensitivity of the method greatly limit its application in clinical diagnosis. Although the virus particles could be detected by electron microscopy, it is not suitable for rapid diagnosis in clinical practice owing to several factors such as being time-intensive and relatively low in the positivity rate. For AP-AAP method, although it could be used to detect viral antigens, non-specific results are usual due to many operating procedures and it is difficult to evaluate the accuracy of the positive results. The detection technology of nucleic acid qualitative PCR is mainly based on fluorescence qualitative polymerase chain reaction. Its key steps involve the effective extraction, isolation and purification of nucleic acid in viruses from a sample, and designing the corresponding specific primer sequence. As a new developed technique, multiple real-time qualitative fluorescent PCR analysis could realize rapid screening of nucleic acid detection and typing of multiple pathogens with strong sensitivity and high specificity by using the technology of hybridization or polymerase chain reaction. Moreover, on the basis of ensuring the sensitivity and specificity of analysis, it could perform detection by micro-sample handling and make operation procedures more easily.

Our results demonstrate that the overall performance of the FPMC analysis method has an overall percent agreement (true-positive and true-negative results) of > 99% for all available targets tested compared with the sequencing method. Discrepancy between the FPMC analysis method and the sequencing method may be due to three main factors. Firstly, the sensitivity of the sequencing method may be low, which will lead to the negative results for those weakly positive samples with CT value being near the cut off of the FPMC analysis method. Secondly, primers of the sequencing method may not be able to cover all sub-types of organisms, and thus some organisms in a sample could not be detected using the sequencing method. Finally, FPMC analysis method is a new assay based on PCR reaction. So there are occasionally false-positive results due to the PCR contamination during the process of experiments. In this study, the performance characteristics of the new FPMC analysis method were evaluated by assessing agreement with the results of the sequencing method, a generally accepted standard method.

However, there are still some limitations in our study. Firstly, this study is lack of another molecular-based method for discordant sample adjudications. Comparisons of this FPMC analysis method with another multiplex panel would provide useful information about discordant results with the sequencing method. But this is beyond the designs of our current study. Secondly, the pathogen spectrum of the FPMC analysis method does not include all pathogens. Therefore, combination of the FPMC analysis method and other molecular methods detecting bacteria could help to improve ability in diagnostic testing of respiratory pathogens. Finally, the lack of detection of influenza A virus and Covid-2019 in this study limits the data on the performance for these targets. In our subsequent research, relevant samples will be collected to elucidate the diagnostic efficacy of the new assay kit for influenza A virus and Covid-19. Overall, the FPMC analysis method is a rapid, accurate, and easy-to-use assay for detection of organisms in clinical specimens from the respiratory tract in clinical laboratories.

### Electronic supplementary material

Below is the link to the electronic supplementary material.

Supplementary Material 1

Supplementary Material 2

## Data Availability

No datasets were generated or analysed during the current study.
