# Supplementary material for: Evaluation of the FPMC respiratory panel for detection of respiratory tract pathogens in nasopharyngeal swab and sputum specimens
Source: Virol J. 2024 Jul 11;21:156. doi: 10.1186/s12985-024-02430-x (PMC11241788; doi:10.1186/s12985-024-02430-x)
Supplement: Supplementary file 1 — Supplementary Material 1 [file 12985_2024_2430_MOESM1_ESM.docx]

|  | **Primer Name** | **Primer Sequence(5'--3')** |
| --- | --- | --- |
| Forward primer | Influenza A virus-F | TCTCATGGAGTGGCTAAAGACA |
| Reverse primer | Influenza A virus-R | TGTTCACTCGATCCAGCCAT |
| Forward primer | Influenza B virus-F | TGGAGAAGGCAAAGCAGAAC |
| Reverse primer | Influenza B virus-R | GACCATCTGCATTTCCCGTC |
| Forward primer | Respiratory syncytial virus-F | AGGTGGGGCAAATATGGAAAC |
| Reverse primer | Respiratory syncytial virus-R | TGTCATGTGTTGGGTTGAGTG |
| Forward primer | Human adenovirus-F | CATCTCGATCCAGCAGACCT |
| Reverse primer | Human adenovirus-R | GATGAGCCGGATCTGACCTG |
| Forward primer | Human metapneumovirus-F | TGTGCGGCAATTTTCAGACA |
| Reverse primer | Human metapneumovirus-R | TTGTARCAAGCAACCARAGC |
| Forward primer | Human bocavirus-F | AACGTCGTCTAACTGCTCCA |
| Reverse primer | Human bocavirus-R | TGCGAGTAGAGTGCCAGTAG |
| Forward primer | *Mycoplasma pneumoniae*-F | AAACTGAACCTCCCCGCTTA |
| Reverse primer | *Mycoplasma pneumoniae*-R | TGGCACTACTTGTAGCTGCT |
| Forward primer | *Chlamydia pneumoniae*-F | GATCCTTGCGCTACTTGGTG |
| Reverse primer | *Chlamydia pneumoniae*-R | GTCTGTTGGCAAGGGGAAAG |
| Forward primer | Human rhinovirus-F | TGAGGCTAGARATTCCCCAC |
| Reverse primer | Human rhinovirus-R | AGAGAAACACGGACACCCAA |
| Forward primer | Coronavirus (229E)-F | GCGTGTTGAAGGTGTTGTCT |
| Reverse primer | Coronavirus (229E)-R | TCTGGGGCCAAAACATTGTG |
| Forward primer | Coronavirus (nl63)-F | GCAGTCGTTCTTCAACTCGT |
| Reverse primer | Coronavirus (nl63)-R | CTGCTCAATGAACTTAGGAAGGT |
| Forward primer | Coronavirus (OC43)-F | GCAACAGAACCCCTACCTCT |
| Reverse primer | Coronavirus (OC43)-R | CGCTGTGGTTTTGGACTCAT |
| Forward primer | Coronavirus (hku1)-F | ACTCCCGGTCATTATGCTGG |
| Reverse primer | Coronavirus (hku1)-R | GAGGCAAAATCGTACCAGGC |
| Forward primer | Parainfluenza virus 1-F | GGCCAAAGATTGTTGTCGAGA |
| Reverse primer | Parainfluenza virus 1-R | GTTGCAGTCTGGGTTTCCTG |
| Forward primer | Parainfluenza virus 2-F | AGCACGGGGTTCCTATGTYA |
| Reverse primer | Parainfluenza virus 2-R | TGCTGCTTTGTGATTGGTGT |
| Forward primer | Parainfluenza virus 3-F | CAGAACCCCGTCCTTAGTGA |
| Reverse primer | Parainfluenza virus 3-R | CACCCAGTTGTGTTGCAGAT |
| Forward primer | Parainfluenza virus 4-F | CAGGCCACATCAATGCAGAA |
| Reverse primer | Parainfluenza virus 4-R | AAGAACGCACTCATTCCGAC |
| Forward primer | Novel coronavirus-19 (N gene)-F | CACCGCTCTCACTCAACATG |
| Reverse primer | Novel coronavirus-19 (N gene)-R | CGTCTGGTAGCTCTTCGGTA |
| Forward primer | Novel coronavirus-19 (ORF1ab gene)-F | GGTGCTTGCATACGTAGACC |
| Reverse primer | Novel coronavirus-19 (ORF1ab gene)-R | ATCACAACCTGGAGCATTGC |

**Table S1 Primer sequences of Sanger sequencing.**
