# Supplementary material for: Evaluation of the FPMC respiratory panel for detection of respiratory tract pathogens in nasopharyngeal swab and sputum specimens
Source: Virol J. 2024 Jul 11;21:156. doi: 10.1186/s12985-024-02430-x (PMC11241788; doi:10.1186/s12985-024-02430-x)
Supplement: Supplementary file 2 — Supplementary Material 2 [file 12985_2024_2430_MOESM2_ESM.docx]

**Table S2 Distribution of multi-organisms combinations in subjects with respiratory infections.**

| **Organism combination detected** | **Total Number** | **No. (NPS)** | **No. (sputum)** |
| --- | --- | --- | --- |
| M.pneumoniae + Adenovirus | 1 | 1 | 0 |
| Human rhinovirus + Respiratory syncytial virus | 2 | 2 | 0 |
| Human rhinovirus + Coronavirus | 7 | 5 | 2 |
| Human rhinovirus + Parainfluenza virus | 9 | 5 | 4 |
| Adenovirus + Respiratory syncytial virus | 1 | 1 | 0 |
| Human rhinovirus + Coronavirus + Parainfluenza virus | 2 | 2 | 0 |
| Coronavirus + Human bocavirus | 1 | 1 | 0 |
| Human rhinovirus + Adenovirus | 3 | 3 | 0 |
| Human rhinovirus + Adenovirus+ Coronavirus | 1 | 1 | 0 |
| Influenza B virus+ Respiratory syncytial virus | 2 | 1 | 1 |
| Parainfluenza virus + Coronavirus | 1 | 1 | 0 |
| Parainfluenza virus + Human bocavirus | 1 | 1 | 0 |
| Respiratory syncytial virus + Human bocavirus | 1 |  | 1 |
| Human rhinovirus + Human bocavirus | 5 | 1 | 4 |
| Parainfluenza virus + Respiratory syncytial virus | 1 |  | 1 |
| M.pneumoniae + Parainfluenza virus | 1 |  | 1 |
| M.pneumoniae + Influenza B virus | 1 |  | 1 |
